# Finding a role for non-coding DNA in trypanosomes

**DOI:** 10.7554/eLife.110271

**Published:** 2026-01-13

**Authors:** Markus R Schmidt

**Affiliations:** 1 Ludwig-Maximilians-Universität München Munich Germany

**Keywords:** *Trypanosoma brucei*, non-coding DNA, repetitive elements, kinetochore, Replication Protein A, cell division, Other

## Abstract

Non-coding DNA is essential for both humans and trypanosomes, despite the large evolutionary divergence between these two species.

**Related research article** Carloni R, Devlin T, Tong P, Spanos C, Auchynnikava T, Rappsilber J, Matthews KR, Allshire RC. 2025. Defining the chromatin-associated protein landscapes on *Trypanosoma brucei* repetitive elements using synthetic TALE proteins. *eLife* **14**:RP109950. doi: 10.7554/eLife.109950.

Whenever we need to perform a task, our cells rely on proteins to accomplish the job. Want to move? That’s a job for actin. Want to see? Rhodopsin will help you. Want to smell? That’s what olfactory receptors do. So, if proteins do everything, why does DNA that does not code for proteins make up such a large part (~98%) of the human genome? Scientists have been working on this question for close to 50 years using various model organisms (Biémont and Vieira, 2006).

Trypanosomes are single-cell parasites that cause sleeping sickness. They are also eukaryotic cells, meaning they are, evolutionarily speaking, closer to plants and animals than to bacteria, despite having diverged from our eukaryotic lineage approximately 500 million years ago. This early divergence makes them an excellent model for studying basic biological concepts: they operate quite differently from humans, yet the fundamental mechanisms of life still need to be performed.

Just like the human genome, trypanosome genomes harbor large non-coding regions, including regions that consist of multiple repeats of short sequences of DNA (Rabuffo et al., 2024). These regions are named after the length of the repeated sequence: the 70 base pair repeat, the 177 base pair repeat, and so on. Moreover, these repeats are found at multiple locations in the genome. Trypanosomes have 11 main chromosomes and hundreds of small chromosomes. The 177 base pair (bp) repeats are found on all of these small chromosomes, although their exact function is unknown (Ersfeld and Gull, 1997).

Now, in eLife, Robin Allshire, Keith Matthews and colleagues at the University of Edinburgh – including Roberta Carloni and Tadhg Devlin as joint first authors – report new insights into the mystery of non-coding DNA in trypanosomes (Carloni et al., 2025). To investigate the role of the 70 bp and 177 bp repeats, Carloni et al. designed so-called TALE proteins that specifically bind to these repeats. Next, they linked the TALE proteins to nearby natural proteins, purified them, and then identified the natural proteins by mass spectrometry.

Many of the natural proteins they identified for 177 bp repeats were components of the kinetochore, a multiprotein complex that is involved in cell division. When a cell divides, it replicates its chromosomes, and a structure called the mitotic spindle pulls the replicated chromosomes apart, ensuring that both daughter cells receive the full set of genetic material (Akiyoshi, 2016). The kinetochore connects the mitotic spindle to a region of the replicated chromosomes called the centromere. Finding the kinetochore at 177 bp repeats is significant because it was not previously known which, if any, sequence would act as the centromere of the smaller chromosomes.

The mitotic spindle is made up of spindle fibers, and kinetochores usually connect the centromere to the ends of these fibers during cell division. However, the large number of small chromosomes found in trypanosomes means that they might outnumber the spindle fibers, so some of them might have to connect to the side of the fiber rather than to the end (Gull et al., 1998; Figure 1). Related to this, the kinetochore contains multiple proteins, and some of these could not be identified at the 177 bp repeats by Carloni et al.: one possible explanation for this is that the composition of the kinetochore is different for smaller chromosomes to enable them to connect to the side of spindle fibers.

**Figure 1.**
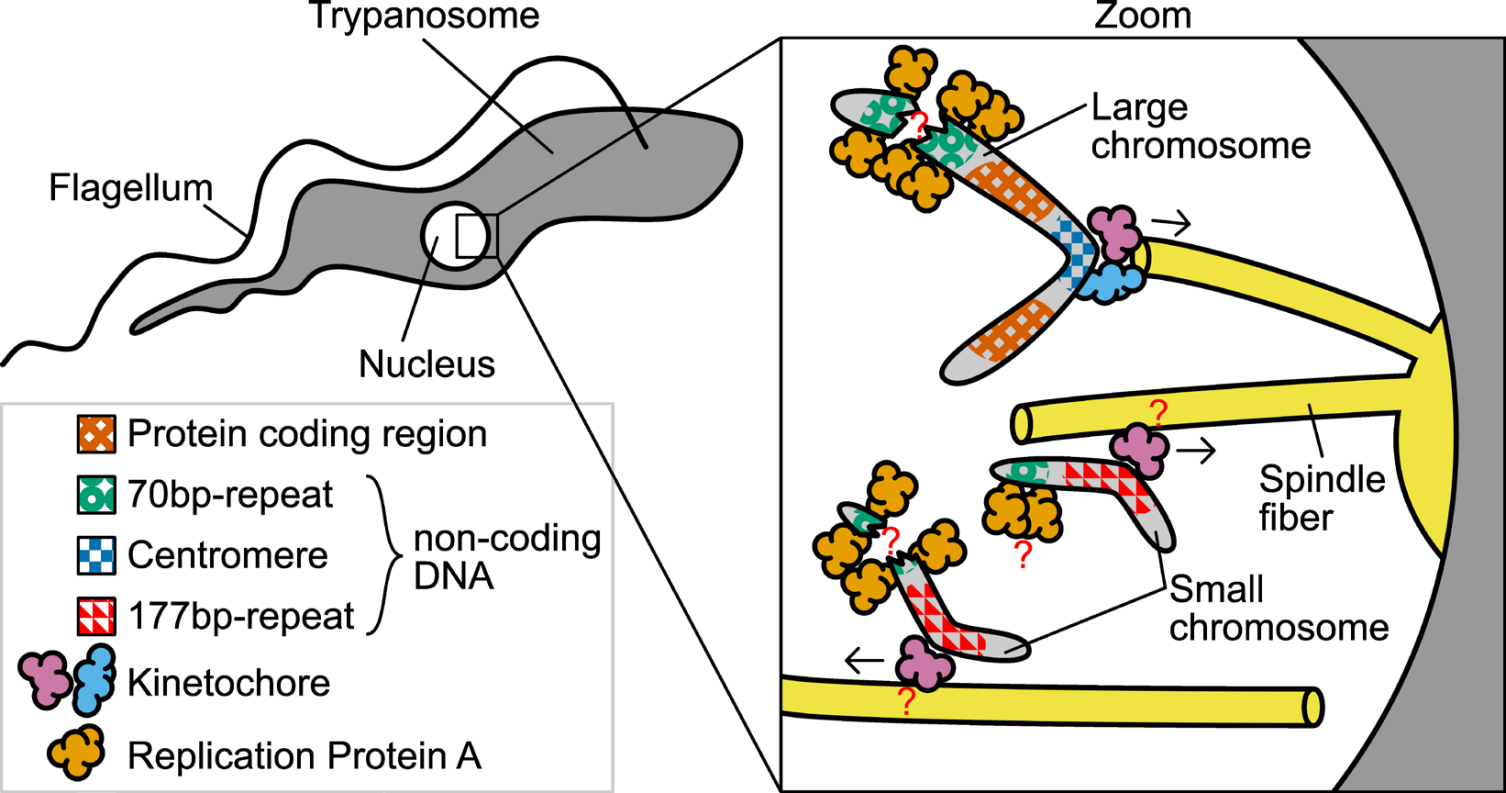
A role for repeat sequences in non-coding DNA of trypanosomes. Top left: Trypanosomes diverged from our eukaryotic lineage about 500 million years ago, making them an excellent model for studying basic biological concepts. Right: Schematic of part of the nucleus of a trypanosome showing one large chromosome (top) and two smaller chromosomes (bottom) being separated by the mitotic spindle (yellow) during cell division. Spindle fibers are attached to the chromosomes via kinetochore proteins (blue and purple), which bind to the centromere (blue squares) of the large chromosome, and to 177 bp repeats (red triangles) in the small chromosomes. Replication Protein A (RPA; orange) was found at 70 bp repeats (green circles), possibly helping to repair DNA breaks. However, it is unclear if RPA is also found at 70 bp repeats where there are no DNA breaks. Components are not drawn to scale.

Carloni et al. also studied 70 bp repeats using the same approach. There, they identified a protein complex called RPA (short for Replication Protein A). RPA usually binds to single-stranded DNA, preventing the DNA from curling up and being degraded, and it has an essential role during the repair of DNA breaks (Dueva and Iliakis, 2020). Trypanosomes exploit such breaks to evade the human immune system. In particular, they periodically switch their ‘surface coat’ by recombining (that is, breaking and reassembling) genomic regions that code for different coat variants (Mugnier et al., 2015; Keneskhanova et al., 2025). This regular switching of coats means that the host immune system never manages to recognize and clear all trypanosomes, which is why trypanosome infections can be lethal if untreated.

70 bp repeats are located near many coat variants, and are expected to break frequently (da Silva et al., 2018), so it is not surprising to find RPA at these locations. However, trypanosomes grown in cell culture switch coats much less frequently than those in the wild (Dreesen and Cross, 2008), so finding RRA at the 70 bp repeats raises interesting questions. Is RPA enrichment at this location really a result of frequent DNA breaks? If there are frequent breaks, why do they not result in coat switches, and is RPA implicated in this?

The work of Carloni et al. brings us a step closer to understanding the purpose of several non-protein-coding regions in trypanosomes. Moreover, it might be possible to apply their approach, as well as their findings, to other organisms.
